# Recommendations from Thai stakeholders about protecting HIV remission (‘cure’) trial participants: report from a participatory workshop

**DOI:** 10.1093/inthealth/ihaa067

**Published:** 2020-11-09

**Authors:** Holly L Peay, Nuchanart Q Ormsby, Gail E Henderson, Thidarat Jupimai, Stuart Rennie, Krittaecho Siripassorn, Kunakorn Kanchawee, Sinéad Isaacson, R Jean Cadigan, Kriste Kuczynski, Udom Likhitwonnawut

**Affiliations:** RTI International, Research Triangle Park, USA; University of North Carolina at Chapel Hill, Department of Social Medicine, USA; University of North Carolina at Chapel Hill, Department of Social Medicine, USA; Center of Excellence in Pediatric Infectious Diseases and Vaccines Faculty of Medicine, Chulalongkorn University, Thailand; University of North Carolina at Chapel Hill, Department of Social Medicine, USA; University of North Carolina at Chapel Hill, Center for Bioethics, USA; Bamrasnaradura Infectious Diseases Institute, Thailand; Center of Excellence in Research on Gender, Sexuality and Health, Faculty of Social Sciences and Humanities, Mahidol University, Thailand; University of North Carolina at Chapel Hill, Department of Social Medicine, USA; University of North Carolina at Chapel Hill, Department of Epidemiology, USA; University of North Carolina at Chapel Hill, Department of Social Medicine, USA; University of North Carolina at Chapel Hill, Center for Bioethics, USA; University of North Carolina at Chapel Hill, Department of Social Medicine, USA; Thailand National CAB on HIV research, Thailand

**Keywords:** cure trials, ethics, HIV, informed consent, stakeholder engagement

## Abstract

**Background:**

The social/behavioral HIV Decision-Making Study (DMS) assesses informed consent and trial experiences of individuals in HIV remission trials in Thailand. We convened a 1-d multi-stakeholder participatory workshop in Bangkok. We provide a meeting summary and reactions from DMS investigators.

**Methods:**

Workshop members viewed de-identified interview excerpts from DMS participants. They deliberated on the findings and made recommendations regarding informed choice for remission trials. Notes and recordings were used to create a summary report, which was reviewed by members and refined.

**Results:**

Workshop members’ recommendations included HIV education and psychosocial support to establish the basis for informed choice, key trial information to be provided in everyday language, supportive decision-making processes and psychosocial care during and after the trial. Concerns included participant willingness to restart antiretrovirals after trial-mandated treatment interruption, unintended influence of the research team on decision-making and seemingly altruistic motivations for trial participation that may signal attempts to atone for stigmatized behavior.

**Conclusions:**

The workshop highlighted community perspectives and resulted in recommendations for supporting informed choice and psychosocial and physical health. These are the first such recommendations arising from a deliberative process. Although some elements are rooted in the Thai context, most are applicable across remission trials.

## Introduction

There is increasing emphasis on involving multiple stakeholders, including affected individuals, advocates and clinicians, in guideline and recommendation development.^1^^,^^2^ We describe a participatory workshop focused on informed choice and protection of HIV remission trial participants. This workshop took place as part of a behavioral and social science research (BSSR) study to assess decision-making for HIV remission trial participation in Thailand, hereafter called the decision-making study (DMS). The DMS is responsive to controversies regarding the ethics of remission (‘cure’) trials,^3^ in particular, trial designs that include interruption of antiretroviral therapy (ART) to assess virologic suppression, typically to evaluate intervention effectiveness.^4^^,^^5^ Potential trial-related risks stem from the experimental intervention and the interruption of highly effective ART and include intervention-related adverse events, symptoms of acute HIV infection, increase of HIV reservoir size, ART resistance and the risk of transmission to sexual partners.^5^^,^^6^ Informed and voluntary choice may be challenging for these complex trials.^7^ In addition, HIV continues to be a highly stigmatized condition in Thailand and elsewhere, necessitating heightened sensitivity regarding protection of vulnerable trial participants.^8^ While BSSR can provide data for addressing ethical controversies related to remission trials,^9^^,^^10^ it is important to involve community stakeholders in determining research implications.

The DMS is led by researchers from the University of North Carolina at Chapel Hill (UNC) and RTI International. It builds on the South East Asia Research Collaboration in HIV (SEARCH010/RV254) clinical research study. SEARCH 010/RV254 (hereafter called ‘SEARCH’) is a long-term observational cohort of individuals enrolled during acute HIV infection in Thailand (clinicaltrials.gov NCT NCT00796146).^11^ SEARCH cohort members receive ART and follow-up care. Four HIV remission trials, all of which included analytic treatment interruption (ATI), have recruited from SEARCH.^12^ DMS investigators interviewed 54 participants and 20 decliners recruited to these remission trials and analyzed the data using rigorous methods.^3^^,^^4^

**Figure 1.**
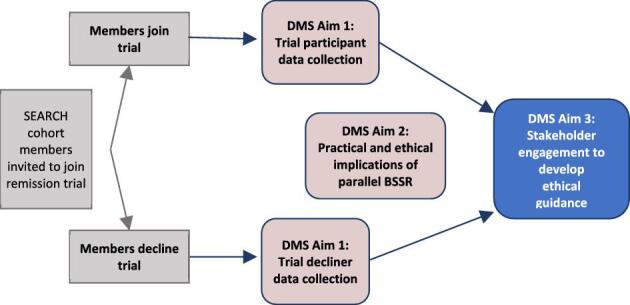
Overview of the decision-making study (DMS) approach and aims.

A DMS overview is shown in Figure 1. The first aim is empirical, to assess motivations to join or decline remission trials and whether and how decision satisfaction changes over time.^4^ The second aim is related to conducting a BSSR study alongside a clinical trial.^13^ The third aim is to create guidance for improving the ethical conduct of HIV remission trials. This paper describes an outcome of the third aim, applying a deliberative process through a participatory workshop to develop recommendations about informed choice and participant protection in remission trials.

## Materials and Methods

We conducted a 1-d participatory workshop in Bangkok, Thailand, in April 2019. The goal was to engage a diverse stakeholder group to critically examine DMS findings and develop recommendations for improving the conduct of future HIV remission trials, particularly trials that include treatment interruption. Workshop members were provided with, and asked to discuss, excerpts of interviews associated with the SEARCH RV405 remission trial.^12^ This randomized, placebo-controlled trial included four doses of experimental vaccine followed by ART interruption. At the time of this workshop, 25 of the 26 RV405 trial participants had experienced viral rebound and were back on ART.

### Participatory workshop members and facilitators

The participatory workshop comprised 12 Thai members invited for their diverse perspectives and cultural knowledge, including CAB members, people living with HIV (PLHIV), medical professionals, social scientists and Thai non-governmental organization representatives (see Additional file 2). The moderator is a well-known and experienced HIV advocate in Thailand. Members were recommended by the moderator and the SEARCH research team. All workshop members were knowledgeable about HIV but their familiarity with remission research varied. None of the workshop members were directly associated (as a clinician or participant) with a SEARCH remission trial.

The meeting facilitators are members of the DMS team: a UNC researcher who is a native Thai speaker (NQO), a BSSR scientist who conducts the DMS interviews (TJ) and the senior BSSR scientist (HLP), who participated via real-time translation.

### Workshop approach

The meeting objectives and processes were developed by DMS investigators with consultation by the Thai-based meeting moderator and SEARCH clinical research team.

Members were provided with de-identified transcript excerpts selected on relevance to the workshop goals. Specifically, these comprised longitudinal interview data from RV405 participants and decliners from the time of consent and trial's end. Interview topics included HIV diagnosis narrative, experience starting and maintaining use of ART, remission trial decision-making, reflections on trial results and decision satisfaction. Interviews with participants included trial experiences such as stopping and restarting ART during the trial.

To create the interview excerpt documents, DMS investigators first selected a subset of participants and decliners to represent a range of decision-making attitudes and experiences. Ultimately, DMS investigators selected excerpts from 4 participants out of 19 and 4 decliners out of 6. Transcripts included both the original Thai and the English translation. Transcript documents were organized into subsections, arranged by timing of the interview and content of the excerpts. Given the geographic proximity of Thai workshop members and interviewees, we removed identifying information including names and other details that we anticipated could lead to re-identification. On the day of the workshop, each member read two documents, one for a trial participant and one for a trial decliner.

### Instructions given to workshop attendees

The workshop members were informed that the meeting had three key objectives:

1. Identify key information that PLHIV need to make an informed trial choice;
2. Use DMS interview data to find support for or against informed choices being made regarding trial participation;
3. Make recommendations about how to ensure that PLHIV can make an informed choice about joining or declining HIV remission trials.

#### Meeting structure

All workshop activities occurred during the 1-d workshop. After introductions and a review of the objectives and processes of the workshop, the meeting followed the organizational structure described below. The meeting was audio-recorded after agreement by the members. The agenda is available as Additional file 1.

### Introduction to SEARCH, SEARCH remission trials and the decision-making study

The Chief of SEARCH provided an overview of the SEARCH research cohort, which comprised individuals diagnosed and treated during the acute phase of HIV infection and the SEARCH remission trials. The Chief of SEARCH provided her perspective on the relevance of the DMS study to provide guidance on the ethical issues that impact participants during remission trial processes. To promote open, candid dialogue, the Chief of SEARCH then left the meeting and no SEARCH investigators or staff were present during the discussion. Next, the DMS aims and processes were described, along with additional detail about the RV405 remission trial. Finally, members were reminded of the sensitive nature of the data presented in the de-identified transcript excerpts and how to maintain the confidentiality of RV405 participants and decliners.

### Discussion regarding making a ‘good choice’ about participating in HIV remission trials

The moderator then led a full-group discussion about what would be needed to make a good choice about participating in an HIV remission trial. Prompts included:

- What does a ‘good choice’ mean for you?
- What type of information is needed?
- How should participants be supported over time? What if they change their mind?

### Review and discussion of transcript excerpts

Workshop members were asked to read their assigned, de-identified participant and decliner interview excerpts. Workshop members were given the following prompt by the moderator: ‘Discuss instances where decision-making and decision satisfaction were good and places with more worries.’ Regarding participant data, they considered: ‘Were there concerning or positive things about participants’ experiences during the trial?’ Workshop members were given time to discuss the data, ask questions and provide immediate reactions.

### Recommendations

The final session was a facilitated discussion to generate specific recommendations for researchers involved in planning and conducting remission trials. The discussion focused on the following question: ‘What do interview data and your own experiences tell you about recommendations to support good decision-making?’

Prompts included:

- What specific types of knowledge/information are needed?
- Who should be involved in decision-making?
- What are signs of good and poor decision-making?
- Are some motivations more concerning than others?

### Postmeeting analysis and reporting

The facilitators took extensive notes, which were reviewed and compared against the workshop audiotape. The facilitators then summarized the themes and recommendations via a consensus process. These results were checked by the moderator. A draft report was prepared, translated into Thai, sent to all workshop members for review and comment then refined. All workshop members were invited to coauthor the manuscript but only three agreed to authorship due to time requirements.

The report is summarized in the Results section. The Discussion section was written as a response by the USA-based DMS investigators, with input from all authors. The final version has been translated to Thai to ensure access for Thai stakeholders.

## Results

### Recommendations regarding overarching and cross-cutting needs

The workshop members identified several cross-cutting needs applicable to SEARCH and the broader Thai HIV population. These concerns were neither reduced nor exacerbated by the DMS interview data. First, workshop members were concerned about the well-being of the SEARCH participants, regardless of enrollment in a clinical trial. They advocated for additional psychological support and stigma reduction efforts, including interventions tailored to the primarily men-who-have-sex-with-men SEARCH cohort. Providing such support was perceived to be the responsibility of the SEARCH and the DMS research teams.

A second cross-cutting need was continuity of treatment, including for participants who may want to withdraw from SEARCH or transition care at study end. Workshop members advocate for participants to be informed of treatment options outside the SEARCH cohort and potential implications of research participation. Regarding the latter, there is a risk that remission trial participation leads to ART resistance and there may be no guarantee of post-trial access to another, equally effective antiretroviral regime. Workshop members felt researchers had a responsibility to assist those in such a situation.

### Recommendations related to potential risks of remission trial participation

Workshop members referenced individual risks of remission trial participation that included ART resistance, HIV symptom progression and increase of HIV reservoir size. They worried that stopping ART temporarily could affect participants’ discipline for taking ART as prescribed. In addition, workshop members considered the risk of transmission to sexual partners during ATI and the complexities of disclosing HIV status and remission trial participation (and thus the need for abstinence or additional protection) to potential sexual partners. The need for education and support about these risks is described further in the remission trial information and support recommendations section.

Workshop members also expressed concerns about potential psychosocial harms, some of which might be exacerbated in the Thai cultural context. For example, they described the potential influence of Kreng Jai, which can be translated as an attitude of respect and consideration for others and an obligation to do the ‘right thing’ in the short term. It involves avoiding conflict or inconvenience of another person, particularly one who is of higher social status.^14^ This may lead participants to defer to the perceived interests of authorities (such as the SEARCH study team) when making decisions.

Potential harms applicable across all cultural contexts included unrealistic trial expectations that could lead to psychological harm if not met during the trial. The members emphasized the importance of understanding participant motivations and discouraging those based on misperceptions, for example, of HIV as a frightening and fatal disease, which may lead participants to see research participation as the only way to escape a terrifying situation. Similarly, workshop members reported the importance of BSSR investigators to longitudinally assess expectations and decision satisfaction during trials and, as necessary, intervene to realign participant expectations with scientific expectations, provide education and/or provide additional psychosocial support.

### Remission trial information and support recommendations

Recommendations fell into three thematic areas. Those related to supportive decision-making center around improving participants’ baseline understanding of HIV, understanding their motivations and goals and enhancing the process of decision-making. Recommendations related to information provision and comprehension refer to trial-related information that should be provided to potential trial participants during the informed consent process. The final set, on providing ongoing support, outlines approaches with which to support participants both during and after remission trial participation.

The recommendations in Table 1 are universally applicable concepts. The members’ discussion, however, also reflected specific challenges related to translation of medical and research concepts. Of note, as in many global settings, there was no consensus regarding the Thai translation of many English-language words that are frequently used in HIV remission research (e.g. ‘remission’, ‘cure’ and ‘HIV reservoir’). Developing the most appropriate and accessible translations of HIV and remission terms and concepts is an emerging issue and one that requires the skill and experience of clinical trial teams, the regulatory bodies reviewing the study materials and the recruiters.

**Table 1.** Recommendations from workshop members on supporting remission trial participants during and after trial decision-making


(1) Supportive decision-making
• To minimize potential influence experienced by individuals considering trial participation, consider alternative recruitment models, for example:
• conducted by someone not a member of the study team, or
• conducted by a research team member along with a peer advocate. Recruiters must be well-informed and experienced.
• Anticipate circumstances that might influence decisions about participation when setting inclusion and exclusion criteria.
• For example, investigators should be sensitive to the timing of recruitment. Someone recently diagnosed with HIV may be emotionally vulnerable and not yet have a solid understanding of living with HIV.
• Employ open-ended questioning to assess trial motivations and discuss thoughts and feelings about trial.
• Work with participants to identify realistic goals.
• Referencing shared goals and progress made toward achieving goals may maintain reasonable expectations and motivate participants to comply with procedures.
(2) Information provision and comprehension
• Potential participants require a baseline understanding of current health status and HIV progression prior to trial decision-making.
• Specific information that should be provided in study materials and consent forms include:
○ Potential harms
○ Potential benefits
○ Study procedures, timeline and anticipated burden/life impact
○ Access to health services during and after the trial
○ Appointment scheduling options
• Key terms such as ‘treatment’, ‘risk’, ‘transmission’ and ‘placebo’ should be clear, straightforward and use everyday terminology.
• Provide anticipatory information relevant to potential impact of participation on participants’ mental health, perceptions of internalized stigma and potential need for support services.
• For studies that include ATI, provide guidance on:
○ what to expect during ATI
○ how participants can take care of their health
○ unanticipated worries that may arise when anticipating viral rebound
○ symptoms that could be experienced
• Information should also be provided on risk of HIV transmission during ATI. This information should be carefully crafted so as not to increase feelings of stigma and guilt.
• Continuity of treatment after joining the study should be addressed.
○ Should treatment resistance or side effects requiring a regimen change occur after ATI, potential participants should be informed about whether that regimen is likely to be available if participants transition to standard clinical settings.
• Researchers should employ open-ended questioning to assess comprehension and offer an opportunity for participants to ask follow-up questions.
(3) Ongoing support
• Regularly follow up with participants to confirm continued understanding of purposes and processes of the study; ask about feelings about study participation.
• Make mental health care and psychosocial support available to study participants and decliners.
• Stopping ART temporarily may later affect participants’ discipline for taking ART as prescribed. Support and monitor poststudy treatment adherence.
• Though trial results should be made available to participants at the end of the trial, researchers should be systematic and thoughtful in their provision of any interim trial results, especially in blinded trials.
• There should be specific, clear commitments that participants will receive necessary care and treatment needed for any unexpected adverse health events that result from study participation.
• Remission studies should ensure and protect the rights of human subjects, including their right to withdraw at any time. Oversight may best be offered by people who are not members of the medical research team.

### Recommendations in response to decision-making study data

After workshop members had reviewed and discussed interview excerpts from RV405 participants and decliners, their initial input was only slightly altered. In most cases, the workshop members felt that participants had sufficient information and made reasonable choices. Members identified examples of information and support needs being met, or not fully met, during education and consent processes and during the trial. Most considered the transcripts to reinforce the need to have additional psychological counseling available based on data from several interviews indicating internalized stigma, lack of disclosure, limited HIV-associated support and/or worries about long-term health outcomes.

Finally, workshop members reacted skeptically to reports in the DMS data about altruism as a primary motivation for participating in potentially risky remission research. Their concern was that what appears as altruism may in fact signal a desire to atone for stigmatized behavior linked to sexual orientation and acquisition of HIV in accordance with the Thai/Buddhist culture's focus on ‘merit-making’. Merit-making is an act of atonement that may lead to uncertain long-term rewards or rewards in the next life. They described this as particularly relevant in Thailand where HIV is still misunderstood as a life-threatening disease. Workshop members felt that research participation should not be used to compensate for perceived moral wrongs and thus this kind of merit-making motivation should be actively discouraged. Instead, any potential for direct medical or incidental benefit was a more acceptable motivation. Rounding out this complex picture, members did understand that the chance of direct medical benefit from the study intervention was very low.

After some deliberation, workshop members came to a consensus that motivations of ‘mutual benefit’ may be the most acceptable and realistic. Mutual benefit would include the potential for short or longer term personal benefit, whether medical or ancillary benefits such as psychological benefits of participating, together with motivations related to improving scientific knowledge. Workshop members ultimately recommended that study teams emphasize the potential for longer term scientific advancements that may benefit the participant and the community, rather than short-term personal medical benefits or merit-making through participation.

## Discussion

### Summary of workshop recommendations to support HIV remission trial participants

**Figure 2.**
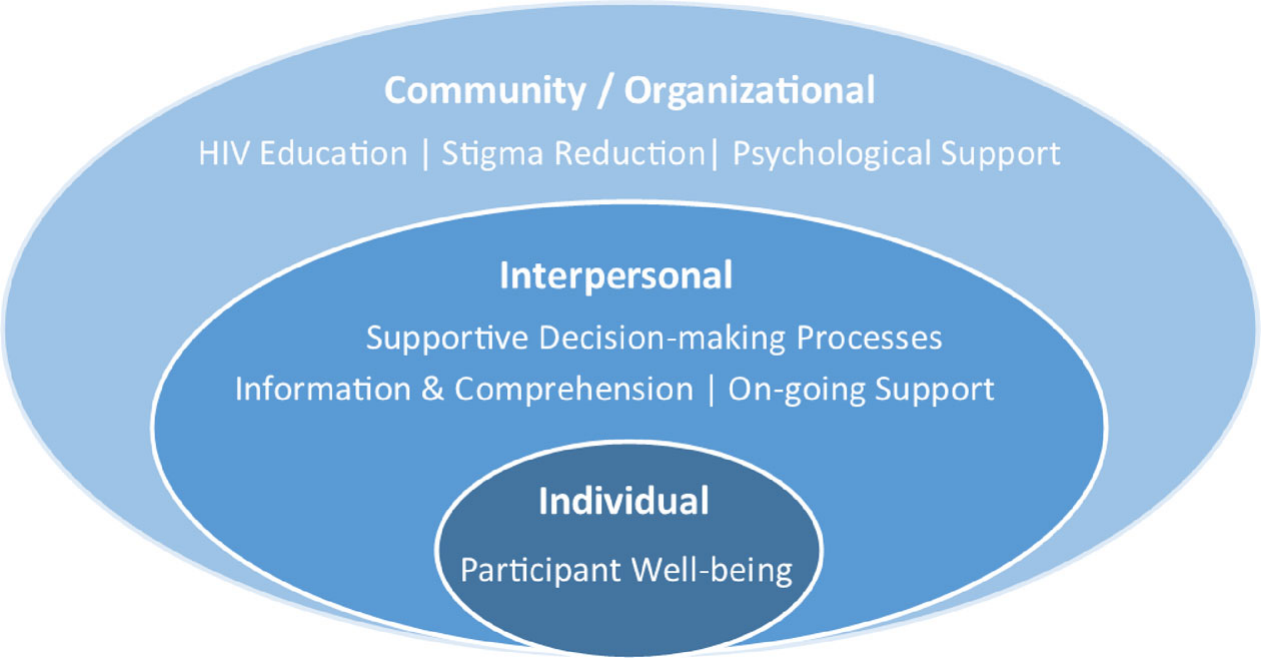
Summary of participatory workshop recommendations: factors to support informed choice for remission trials in the context of individual well-being

The workshop achieved its objectives of engaging in a deliberative process and creating a set of recommendations. Though the workshop objectives focused on supporting informed choice for HIV remission studies, members advocated for a broader range of recommendations. These included: education about HIV and treatment options, stigma reduction efforts and global psychosocial support for all PLHIV to provide a strong backbone upon which trial decisions can be implemented; participant-friendly trial information; anticipatory guidance about the ATI experience and transmission risk; careful recruitment and consenting approaches that include exploration of participant motivations and assessment of comprehension; and ongoing efforts to assess and enhance the well-being of participants in HIV remission trials (Figure 2).

The workshop recommendations were framed in response to members’ perception of risks of remission trial participation that included medical and psychosocial risks to participants and to sexual partners. Since these risks are universal to HIV remission trials, the associated recommendations should be broadly applicable. A few of their concerns (e.g. the importance of merit-making in the Buddhist culture) are culturally rooted and may be less applicable to other settings.

Further, our workshop recommendations overlap with two recent recommendation-focused publications.^15^^,^^16^ The 2018 TAG Community Recommendations report is supported by data from advocates and the 2019 report by Julg et al. stems from an interdisciplinary consensus meeting in the USA, primarily scientists along with several PLHIV and advocates. These reports attend to the acceptability of employing ATI trials and technical aspects, such as design and inclusion criteria, as well as some recommendations on the protection of human subjects that are consistent with ours. The TAG report indicated high concern about transmission risk and developing resistance and HIV progression during ATI; and additional, less commonly reported concerns about the mental health of participants, overly optimistic trial expectations, anxiety during ATI when anticipating viral rebound, the need for regular feedback from study participants and the need for risk counseling and support for those who become detectable.^15^ Julg et al.’s recommendations also consider both personal health risks and transmission risk and the need for careful informed consent and monitoring of participants’ psychosocial experience during an ATI trial. The group also recommended the addition of BSSR studies into HIV remission protocols with ATI.^16^

### Response to the workshop recommendations by the authoring group

This section reflects the further deliberation on the participatory workshop results by the authoring group, which includes the decision-making study investigators and three Thai workshop members. The authoring group endorses the workshop recommendations with the understanding that national, regional and local regulations and sociomedical context will make some of the specific recommendations more or less applicable or feasible.

It was reassuring that DMS interview excerpts did not cause major concerns about how cohort members made decisions whether to join or decline trial participation. This matched the DMS study team's interpretation. The workshop recommendations primarily arose out of the diverse stakeholders’ own experiences and expertise, although members found support for their recommendations within the DMS study data. The notable exception was the concern about altruistic motivations, which arose primarily from the DMS study data.

The workshop left the DMS research team with four issues to consider. First, the need for additional counseling and support for PLHIV was consistently reported. Members felt that DMS data reflected this need to a higher degree than the DMS investigators did. What might account for this difference in perception? Workshop members, as local stakeholders, are more familiar with the social contexts of Thai PLHIV and may thus start from a different set of preconceived notions about the challenges faced by participants, independent of their involvement (or not) in remission trials. It is also possible that participants downplayed their psychosocial challenges due to social desirability bias, Kreng Jai-influenced concerns about reporting negative perceptions of SEARCH or as a matter of Thai reservedness, thus making it less obvious to outside researchers. It may be that the process of excerpting interviews retained more problematic components of the interviews while removing more positive or benign interview content, resulting in a different overall interpretation for workshop members. In any case, it is reasonable to recommend implementation of pilots for enhanced mental health services to be offered through the SEARCH cohort. Currently available services include counseling, a social/support club and psychiatric services for those who are referred by the SEARCH clinicians.

Second, workshop members raised concerns about the unintended influence of the SEARCH team on trial decision-making, especially given the Thai culture and influence of Kreng Jai. DMS data indicate that belonging to SEARCH influenced decisions of some individuals recruited to remission trials.^3^^,^^4^ For many, close relationships developed in the context of trusted care provision from the SEARCH investigators. We observed that several trial decliners worried about appearing ‘selfish’, perhaps violating social norms of harmony and accommodation. Bechtel and Apakupakul wrote about the disruption of Kreng Jai in the context of HIV/AIDS in southern Thailand: ‘Conflicts are unacceptable in Thai culture as harmony and conformance are to be maintained to the greatest extent possible.’^17^ These underlying cultural forces are important to consider in assessment of decision-making for research participation. The potential for investigator influence, both positive and negative, have been identified in many other contexts, making this a universal area of concern.

Third, workshop members’ views about altruism gave rise to a complicated set of issues. The first is the potential impact of internalized stigma on decision-making for trial participation. Research participation driven by internalized stigma challenges the ideal of voluntary choice. Goffman defines stigma as reducing the individual in question ‘from a whole and usual person to a tainted, discounted one.’^18^ Internalized stigma results in an individual applying stigmatizing stereotypes to him/herself and accepting them as valid.^19^ It is a multifaceted phenomenon that tends to be overlooked as a potential influence on trial decision-making, where one's negative self-concept may lead one to have a lower stake in protecting one's health and well-being. This will be further explored in our subsequent DMS data collection. Prior research, including in Thailand, has shown a positive impact of stigma reduction efforts on both institutional^20^ and internalized^21^ stigma. Future research might address whether stigma reduction interventions improve the capacity for voluntary informed choice.

Further, the voluntary nature of research participation could be compromised if participants joined trials to ‘make merit’ to atone for their sexuality or HIV status. The proportion of research participants who are motivated in this questionable way is unknown. If merit-making is a widespread motivation for action in the Thai context, might there also be merit-making motivations untainted by stigma? Regardless, it may be unreasonable to discourage all such motivations. In future analyses we will attempt to further differentiate motivations associated with stigma, atonement and Kreng Jai. Without the workshop, these complexities and nuances would not have been raised.

Fourth, workshop members were highly supportive of BSSR conducted alongside remission trials to assess psychosocial needs, trial motivations and outcomes such as willingness to restart ART after treatment interruption. Workshop members, however, raised questions about the obligations of BSSR investigators. For scientists who conduct in-depth interviews, is it acceptable to simply collect data as observers? Or do we have an obligation to provide supportive and educational services, in the same manner that workshop members perceived the SEARCH research team to have such obligations? These challenges are not unique to our setting; historical^22^ and more recent^23^^,^^24^ studies examine the obligation of BSSR investigators to subjects. Researcher obligation, the potential need to integrate ancillary care into the research encounter and the boundaries of such care (i.e. directly related to the study experience or in response to more global needs) are areas for ongoing consideration.

### Limitations

Our approach of providing workshop members with de-identified interview excerpts was required for practicability and confidentiality. It is unclear how workshop members’ reactions may have differed if full interview transcripts were shared, that is, whether they would be reassured further or if they might identify other areas of concern. Workshop members read transcripts in the original Thai while the analysis was conducted using English translations. This may have contributed to different interpretations. Though the workshop facilitators, the moderator and investigators have diverse identities, it is inevitable that positionality influenced the authors’ perspectives and interpretations of the workshop.

### Conclusions

This participatory workshop advanced understanding of stakeholder perspectives on HIV remission research through deliberative engagement of members with knowledge stemming from experience and formal and informal instruction. It resulted in recommendations for supporting informed choice and mental and physical health. Although there are elements to the recommendations rooted in the specific Thai context, many conceptually overlap with prior recommendations^15^^,^^16^ and are universally applicable. Our recommendations are unique in having arisen through a deliberative, stakeholder-engaged process. While a range of stakeholders were included in the process, additional engagement efforts to nuance and update recommendations would be useful.

Additional research should further assess participation motivations as well as anticipated and experienced risks, spanning physical, psychosocial and transmission risks. Such data are vital to foster nuanced recommendations over time. In addition, this evidence base informs the implementation and evaluation of interventions to support decision-making and the well-being of PLHIV, those considering remission trial participation, their partners who share trial-related risks and/or the broader community impacted by HIV remission research.

## Supplementary data

Supplementary data are available at International Health online.

## Supplementary Material

ihaa067_Supplemental_File
