# Supplementary material for: Recommendations from Thai stakeholders about protecting HIV remission (‘cure’) trial participants: report from a participatory workshop
Source: Int Health. 2020 Nov 9;12(6):567–74. doi: 10.1093/inthealth/ihaa067 (PMC7650909; doi:10.1093/inthealth/ihaa067)
Supplement: ihaa067_Supplemental_File [file ihaa067_supplemental_file.zip › Appendix_workshop_Peay.docx]

**Appendix 1: Workshop Agenda**

| **Workshop on Decision Making for HIV Remission Trials in Bangkok** |
| --- |
| Welcome  By Dr.Nittaya Phanuphak Pungpapong **(SEARCH)** and Dr. Holly Peay (RTI International) |
| Opening the workshop  By Udom Likhitwonnawut (Moderator) |
| Introduce HIV remission trials at SEARCH  By Dr. Nittaya Phanuphak Pungpapong (SEARCH)  Introduce the Decision Making Study and the clinical trial RV405  By Thidarat Jupimai (Chulalongkorn University) |
| Full group discussion: What is required to be able to make a good choice about participating in an HIV remission trial?  By Udom Likhitwonnawut (Moderator) |
| Read excerpts from DMS interviews; Questions and answers |
| Full group discussion: Response to interview excerpts  By Udom Likhitwonnawut (Moderator) |
| Full group discussion: Developing recommendations for researchers  By Udom Likhitwonnawut (Moderator) |
| Summary and closure |

**Appendix 2: List of Participants**

**Patchara Charuthamrong,** Nurse Educator**,** Armed Forces Research Institute of Medical Sciences

**Kunakorn Kanchawee,** Researcher, Mahidol University

**Apiwat Kwangkaew,** National Community Advisory Board Member /Mercy /TNP+

**Chutamanee Mullawong,** Research assistant, Center of Excellence in Pediatric Infectious Diseases and Vaccines Faculty of Medicine, Chulalongkorn University

**Somchai Phromsombat,** Director, The Poz Home Center Foundation

**Rotjana Piyabanharn,** Nurse Educator, Armed Forces Research Institute of Medical Sciences

**Lawan Sarovat,** National Community Advisory Board Member, Thai NGO Coalition on AIDS, Retro CAB

**Krittaecho Siripassorn,** Physician, Bamrasnaradura Infectious Diseases Institute, DDC, MOPH, Thailand

**Jirawat Suksamosorn,** Representative, CDC – Silom Community Clinic at Tropmed

**Nimit Tienudom,** Director, AIDS Access Foundation

**Kamon Uppakaew,** Representative, The Thai Network of People Living with HIV/AIDS, AIDS Foundation

**Prisna Worraharn,** Research assistant, Center of Excellence in Pediatric Infectious Diseases and Vaccines Faculty of Medicine, Chulalongkorn University

**Meeting team:**

**Udom Likhitwonnawut,** Thai National Community Advisory Board Member ***(Moderator)***

**Thidarat Jupimai,** Social Scientist, Center of Excellence in Pediatric Infectious Diseases and Vaccines Faculty of Medicine, Chulalongkorn University

**Nuchanart Q Ormsby,** Researcher, Department of Social Medicine, University of North Carolina at Chapel Hill

**Holly Peay,** Senior Researcher, Center for Newborn Screening, Ethics, and Disability Studies, RTI International

**Pratompong Serkpookiaw**, Translator
